# Increased concentration of serum TNF alpha and its correlations with arterial blood pressure and indices of renal damage in dogs infected with *Babesia canis*

**DOI:** 10.1007/s00436-014-3792-1

**Published:** 2014-02-20

**Authors:** Wojciech Zygner, Olga Gójska-Zygner, Piotr Bąska, Ewa Długosz

**Affiliations:** 1 Division of Parasitology, Department of Preclinical Sciences, Faculty of Veterinary Medicine, Warsaw University of Life Sciences, Ciszewskiego 8, 02-786 Warsaw, Poland; 2 Multiwet Small Animal Health Clinic, Gagarina 5, 00-753 Warsaw, Poland; 3 Division of Pharmacology and Toxicology, Department of Preclinical Sciences, Faculty of Veterinary Medicine, Warsaw University of Life Sciences, Ciszewskiego 8, 02-786 Warsaw, Poland

## Abstract

Canine babesiosis is a tick-borne disease caused by parasites of the genus *Babesia*. Tumour necrosis factor alpha (TNF-α) is a cytokine that plays a role in the pathogenesis of canine babesiosis. In this study, the authors determined the concentration of serum TNF-α in 11 dogs infected with *Babesia canis* and calculated Spearman’s rank correlations between the concentration of TNF-α and blood pressure, and between TNF-α and indices of renal damage such as: fractional excretion of sodium (FE(Na^+^)), urinary creatinine to serum creatinine ratio (UCr/SCr), renal failure index (RFI), urine specific gravity (USG) and urinary protein to urinary creatinine ratio (UPC). The results demonstrated statistically significant strong negative correlations between TNF-α and systolic arterial pressure (*r* = −0.7246), diastolic arterial pressure (*r* = −0.6642) and mean arterial pressure (*r* = −0.7151). Serum TNF-α concentration was also statistically significantly correlated with FE(Na^+^) (*r* = 0.7056), UCr/SCr (*r* = −0.8199), USG (*r* = −0.8075) and duration of the disease (*r* = 0.6767). The results of this study show there is an increase of serum TNF-α concentration during canine babesiosis, and the increased TNF-α concentration has an influence on the development of hypotension and renal failure in canine babesiosis. This probably results from the fact that TNF-α is involved in the production of nitric oxide and induction of vasodilation and hypotension, which may cause renal ischaemia and hypoxia, and finally acute tubular necrosis and renal failure.

## Introduction

Canine babesiosis is a protozoan disease caused by the parasites of the genus *Babesia* (Matijatko et al. 2012). In Europe, canine babesiosis can be caused by infection with species such as *Babesia canis*, *Babesia vogeli* and *Babesia gibsoni*. Moreover, two species, *Babesia felis*-like and *Babesia microti*-like (now considered as *Theileria annae*), were detected in Germany in dogs imported from Romania, and in Spain, respectively (Hamel et al. 2012; Matijatko et al. 2012). Among these *Babesia* species, only *B. canis* has been detected in Poland (Adaszek and Winiarczyk 2011).

Tumour necrosis factor alpha (TNF-α) is a proinflammatory cytokine that plays a significant role in the pathogenesis of babesiosis and malaria, and influences the severity of these diseases (Hemmer et al. 2000; Krause et al. 2007). Complications of canine babesiosis, such as multiple organ dysfunction syndrome and hypotension may result from the parasite, the host or parasite-host interactions (Matijatko et al. 2012). It is probable that TNF-α is involved in the development of hypotension, renal dysfunction and septic shock in canine babesiosis (Matijatko et al. 2009).

Parameters such as fractional excretion of sodium (FE(Na^+^)), urinary creatinine to serum creatinine ratio (UCr/SCr), renal failure index (RFI), urine specific gravity (USG) and urinary protein to urinary creatinine ratio (UPC) are useful in the differentiation between prerenal and renal azotaemia in azotaemic dogs (Stockham and Scott 2008; Waldrop 2008; Lefebvre 2011).

The purpose of this study was to determine correlations between serum TNF-α concentration and indices of azotaemia (serum urea and creatinine concentration), renal failure indices and blood pressures in dogs infected with *B. canis*.

## Materials and methods

**Fig. 1:**
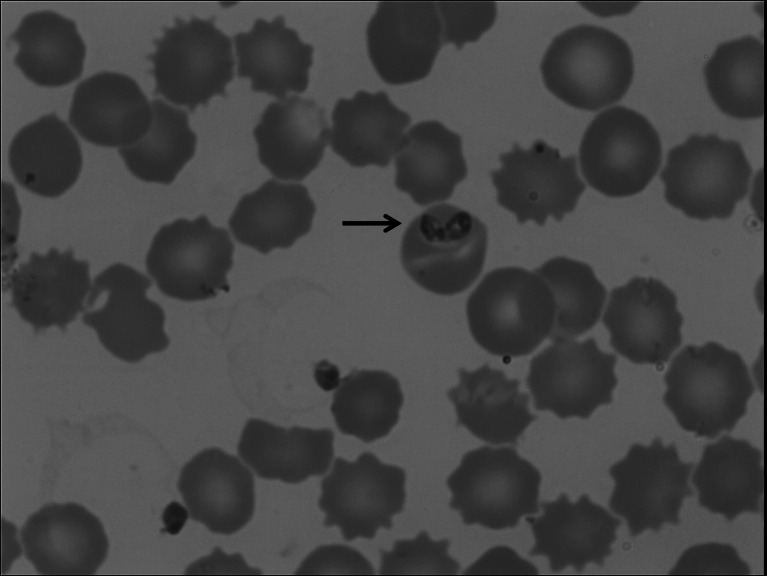
Two *B. canis* merozoites (*arrow*) within red blood cell observed in blood smear (May-Grünwald-Giemsa staining)

Blood, serum and urine samples were collected from 11 dogs of various breeds (Table 1) infected with *B. canis* prior to treatment. Diagnosis of infection with *B. canis* was based on the results of a blood smear examination (Fig. 1) and confirmed by the PCR method described in previous work (Zygner et al. 2012a). Eight clinically healthy dogs were used as a control group. Clinical examination, including blood pressure measurement, was performed during the first visit to the clinic, before sample collection and treatment. Systolic arterial pressure (SAP), diastolic arterial pressure (DAP), mean arterial pressure (MAP) and pulse pressure (PP) were determined using the non-invasive oscillometric technique (Cardell veterinary monitor 9405, Midmark, USA). The concentration of TNF-α in serum samples was determined using an ELISA kit (Quantikine ELISA Canine TNF-α, R&D Systems) and read by an ELISA reader at wavelength 450 nm (MRX Microplate Reader, Dynatech Laboratories) using the program Revelation (version 4.25). Creatinine concentrations (in serum and urine), serum urea concentration and urinary protein concentration were determined by a clinical chemistry analyser (XL 640, Erba Mannheim, Germany). Serum and urinary sodium concentrations were also determined using a clinical chemistry analyser (MEDICA Easy Electrolytes, the Netherlands). Prior to determination of the sodium concentration, urine samples were centrifuged (2,000 rpm, 5 min), then diluted tenfold in deionized water. Urine specific gravity (USG) was determined using a veterinary refractometer (Reichert VET 360, Reichert, USA). The obtained results allowed calculation of indices of renal damage such as FE(Na^+^), UCr/SCr, RFI and UPC. The FE(Na^+^) and RFI were calculated using the following formulas: FE(Na^+^) = UNa × SCr × 100 % ÷ SNa × UCr and RFI = UNa × SCr ÷ UCr, respectively; where UNa is the urinary sodium concentration, SCr is the serum creatinine concentration, SNa is the serum sodium concentration and UCr is the urinary creatinine concentration (Waldrop 2008). The results were analysed using the program Statistica 10. The Mann–Whitney *U* test was used to compare serum TNF-α concentrations between 8 healthy dogs (group A) and 11 dogs infected with *B. canis* (group B). Spearman’s rank correlation coefficient was used to calculate correlations between serum TNF-α concentrations in dogs infected with *B. canis* and their blood pressures (SAP, DAP, MAP and PP), serum urea and creatinine concentrations, FE(Na^+^), UCr/SCr, RFI USG, UPC and the duration of the disease before admission to the clinic. The value of *p* < 0.05 was considered significant.

**Table 1:** Individual values of serum TNF-α concentrations, blood pressure and renal indices, and clinical signs in 11 dogs with babesiosis

Parameter	I	II	III	IV	V	VI	VII	VIII	IX	X	XI
TNF-α	20.1	18.7	0	14.7	0	0	27.8	0	26.5	15.5	0
SAP	83	87	120	118	125	121	117	115	70	81	127
DAP	55	44	90	72	87	72	62	60	52	45	86
MAP	74	73	110	103	112	105	99	97	64	69	113
PP	28	43	30	46	38	49	55	55	18	36	41
SCr	4.6	5.1	1.1	1	1	1.2	1.7	1	2.8	3.3	1
SUr	462	264	39	42	38	32	44	44	166	181	42
UCr	52	15.6	161	40	128	281	60	528	75	100	169
UNa	151	168	84	97	65	57	19	108	39	12	71
SNa	141	137	140	137	139	142	146	145	151	146	144
FE(Na^+^)	9.47	40.09	0.41	1.77	0.36	0.17	3.69	0.14	0.96	0.27	0.29
UCr/SCr	11.3	3.1	146.4	40	128	234.2	35.3	528	26.8	30.3	169
RFI	13.3	54.9	0.6	2.4	0.5	0.2	0.5	0.2	1.5	0.4	1.7
USG	1.013	1.014	1.039	1.060	1.045	1.042	1.005	1.050	1.013	1.016	1.040
UP	160	0	0	520	2250	345	0	1128	3235	820	0
UPC	3.08	0	0	13	17.58	1.23	0	2.14	43.13	8.2	0
DD	4	3	2	1	1	1	2	1	3	2	2
CS	An, Ap	An, Ap, S, F, Tc, Tp	An, Ap, F, V	DA, Ap, F, Tc, Tp, DBU	An, Ap, F, Tc, Tp, DBU	An, Ap, F	An, Ap, F, Tc, Tp	DA, Ap, F	An, Ap, F, DBU	An, Ap, F, V	An, Ap
DS	D, FM, 10y	SBD, FM, 7y	GSc, M, 6y	JRT, M, 3y	MBD, FM, 8y	MBD, FM, 4y	MBD, M, 2y	H, FM, 6y	GSh, M, 1y	GR, FM, 11y	MBD, FM, 3y

*I* to *XI* numbering of dogs, *TNF-α* tumour necrosis factor (in picograms per milliliter), *SAP* systolic arterial pressure (in millimeters of mercury), *DAP* diastolic arterial pressure (in millimeters of mercury), *MAP* mean arterial pressure (in millimeters of mercury), *PP* pulse pressure (in millimeters of mercury), *SCr* serum creatinine concentration (in milligrams per deciliter), *SUr* serum urea concentration (in milligrams per deciliter), *UCr* urinary creatinine concentration (in milligrams per deciliter), *UNa* urinary sodium concentration (in milliequivalents per liter), *SNa* serum sodium concentration (in milliequivalents per liter), *FE(Na* ^*+*^ *)* fractional excretion of sodium (in percent), *UCr/SCr* urinary creatinine to serum creatinine ratio, *RFI* renal failure index, *USG* urine specific gravity, *UP* urinary protein concentration (in milligrams per deciliter), *UPC* urinary protein to urinary creatinine ratio, *DD* duration of the disease (in days), *CS* clinical signs, *DS* description of the dog (breed, sex and age in years), *An* anorexia, *Ap* apathy, *S* seizures, *F* fever, *Tc* tachycardia, *Tp* tachypnoea, *V* vomiting, *DA* decreased appetite, *DBU* dark-brown urine, *FM* female, *M* male, *D* Dachshund, *SBD* St. Bernard dog, *GSc* Giant Schnauzer, *JRT* Jack Russell Terrier, *MBD* mixed breed dog, *H* Hovawart, *GSh* German Shepherd, *GR* Golden Retriever

## Results

**Fig. 2:**
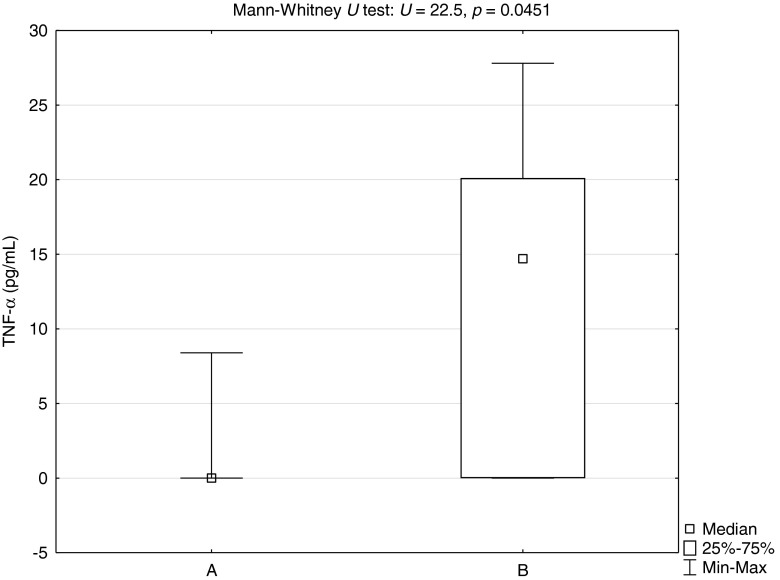
Comparison of TNF-α concentrations between 8 healthy dogs (group A) and 11 dogs infected with *B. canis* (group B)

The individual results of 11 dogs infected with *B. canis* are presented in Table 1. Apathy, anorexia and fever were the most prevalent clinical signs. Detectable concentrations of TNF-α were present in the serum of 6 of the 11 infected dogs. The median serum TNF-α concentration in these dogs amounted to 14.7 pg/mL. Comparison of TNF-α concentrations between groups A and B showed a statistically significantly higher median in infected dogs (Fig. 2). There were statistically significant negative correlations between TNF-α concentration and blood pressures (SAP, DAP and MAP); however, there was no correlation between PP and TNF-α (Table 2). Statistically significant positive correlations were observed between TNF-α concentration and serum urea, serum creatinine and FE(Na^+^) (Table 2). Statistically significant negative correlations between TNF-α concentrations and UCr/SCr ratio and USG were also observed. There were no statistically significant correlations between TNF-α and RFI or UPC. Duration of the disease was statistically significantly correlated with serum TNF-α concentration (Table 2).

**Table 2:** Spearman’s rank correlation between concentration of TNF-α in serum of 11 dogs infected with *B. canis* and blood pressures, serum urea, creatinine and other renal indices

Correlation		*r*	*p*
TNF-α	SAP	−0.7246	0.0116*
	DAP	−0.6642	0.0258*
	MAP	−0.7151	0.0134*
	PP	−0.1911	0.5841
	SUr	0.7232	0.0119*
	SCr	0.7075	0.0149*
	FE(Na^+^)	0.7056	0.0153*
	UCr/SCr	−0.8199	0.0019*
	RFI	0.3927	0.2322
	USG	−0.8075	0.0026*
	UPC	0.1219	0.7209
	DD	0.6767	0.0222*

*TNF-α* tumour necrosis factor alpha, *SAP* systolic arterial pressure, *DAP* diastolic arterial pressure, *MAP* mean arterial pressure, *PP* pulse pressure, *SUr* serum urea concentration, *SCr* serum creatinine concentration, *FE(Na* ^*+*^ *)* fractional excretion of sodium, *UCr/SCr* urinary creatinine to serum creatinine ratio, *RFI* renal failure index, *USG* urine specific gravity, *UPC* urinary protein to urinary creatinine ratio, *DD* duration of the disease (days), *r* Spearman’s rank correlation coefficient, *p* a value of *p*

^a^Statistically significant result

## Discussion

This study showed increased TNF-α concentrations in the sera of dogs infected with *B. canis*. A similar result has been observed in dogs experimentally infected with *Rangelia vitalii*, which is a protozoan parasite that causes canine piroplasmosis in Brazil (Da Silva et al. 2013; Paim et al. 2013). Increase of serum TNF-α concentration has also been observed in human and bovine babesiosis (Shaio and Lin 1998; Kontaş and Salmanoğlu 2006).

The authors of this research detected a strong negative correlation between increased serum levels of TNF-α and blood pressure. This may result from the fact that TNF-α is involved in the production of nitric oxide and induction of vasodilation and hypotension (Jacobson et al. 2002; Chauvin et al. 2009). In a previous study, Zygner and Gójska-Zygner (Association between decreased blood pressure and azotaemia in canine babesiosis. Pol J Vet Sci, accepted for publication) showed a negative correlation between blood pressure and the concentration of blood urea and creatinine, as well as lower blood pressure in azotaemic dogs infected with *B. canis* in comparison to non-azotaemic dogs with canine babesiosis. Thus, it seems probable that the observed strong correlation in this study between serum levels of TNF-α and renal indices such as FE(Na^+^) and UCr/SCr ratio result from the influences of decreased blood pressure and/or TNF-α on renal failure in canine babesiosis. In previous research, Tracey et al. (1987) showed that injection of dogs with a high dose of TNF-α led to acute tubular necrosis. Increase of the FE(Na^+^) and RFI and decrease of the UCr/SCr ratio are indicative for acute tubular necrosis (Waldrop 2008); such changes have previously been observed in dogs infected with *B. canis* (Zygner et al. 2012b, 2013). However, in this study, the RFI was not correlated with TNF-α. This might result from the small group of dogs examined and is an area that requires further research.

The observation in this study of a strong negative correlation between the level of serum TNF-α and USG may also result from an increase in the severity of renal failure, or alternatively, a change from pre-renal azotaemia (higher USG) into renal azotaemia (lower USG) with a concurrent increase of the serum TNF-α concentration. In this work, all azotaemic dogs had relatively low USG (1.013–1.016), but not isosthenuria. One dog had hyposthenuria (USG, <1.008), and serum levels of urea and creatinine were at the upper reference intervals in this dog; thus, it cannot be excluded that azotaemia was developing in this dog. It should also be mentioned that the USG value (decreasing) can be influenced by other factors such as cortisol or aldosterone (Watson 1998); increases in plasma cortisol concentrations have been observed in dogs with canine babesiosis caused by *Babesia canis rossi* (Schoeman et al. 2007). Moreover, previous work has indicated that the hyponatraemia, hypokalaemia and increased fractional excretions of sodium and potassium observed during canine babesiosis may be connected to an increase of aldosterone during the disease (Adaszek et al. 2012; Zygner et al. 2012a, 2012b).

There was no statistically significant correlation between the TNF-α concentration and UPC in this study. Lobetti and Jacobson (2001) observed a correlation between the value of UPC and severity of babesiosis caused by *B. rossi*. In theory, UPC is a useful tool in clinical recognition of damage of renal glomeruli when increasing above values of 0.5–1.0 in azotaemic dogs (Stockham and Scott 2008). However, damage of renal glomeruli, although mentioned by Wozniak et al. (1997) in experimentally infected dogs with *B. gibsoni*, has been rarely observed and mild in dogs with babesiosis (Irwin and Hutchinson 1991; Máthé et al. 2007). In this study, increase of UPC was observed in 7 out of 11 dogs infected with *B. canis*. However, in these dogs, haemoglobinuria and/or bacteriuria were detected which might influence the protein concentration in urine. The observed lack of correlation between UPC and TNF-α concentration in this research is in agreement (provided decreased blood pressure results from increased TNF-α concentration) with the results of previous work in which a lack of a correlation between UPC and decrease of blood pressure was observed (Buranakarl et al. 2007).

In this work, there were strong positive correlations between serum TNF-α concentration and concentrations of serum urea and creatinine, which suggested that cytokine has an influence on the development of azotaemia in canine babesiosis. Additionally, the correlation between duration of the disease and TNF-α concentration suggested that this cytokine is involved in the development of pre-renal azotaemia, caused by hypotension, at the beginning of the disease and further, is involved in renal azotaemia caused by renal hypoxia when the disease is progressing.

The results of this study show there is an increase in the serum TNF-α concentration in canine babesiosis and that the increased TNF-α concentration influences the development of hypotension and renal failure in canine babesiosis. A previous study has shown that the severity and outcomes of babesiosis in mice experimentally infected with human babesial strains of *B. microti* and *Babesia duncani* depend on the host’s immune response and that increased production of TNF-α plays an important role in the pathogenesis of the disease (Hemmer et al. 2000). The results of this work allow us to suppose that this cytokine may also have an influence on the severity of babesiosis in dogs. Further work examining the influence of TNF-α on other complications observed in canine babesiosis are needed to understand the role of this cytokine in the pathogenesis of this disease.
